# Black race as a predictor of poor health outcomes among a national cohort of HIV/AIDS patients admitted to US hospitals: a cohort study

**DOI:** 10.1186/1471-2334-9-127

**Published:** 2009-08-11

**Authors:** Christine U Oramasionwu, Jonathan M Hunter, Jeff Skinner, Laurajo Ryan, Kenneth A Lawson, Carolyn M Brown, Brittany R Makos, Christopher R Frei

**Affiliations:** 1 College of Pharmacy, The University of Texas, Austin, TX, USA; 2 Department of Medicine, The University of Texas Health Science Center, San Antonio, TX, USA; 3 The National Institute of Allergy and Infectious Diseases, The National Institutes of Health, Bethesda, MD, USA

## Abstract

**Background:**

In general, the Human Immunodeficiency Virus/Acquired Immunodeficiency Syndrome (HIV/AIDS) population has begun to experience the benefits of highly active antiretroviral therapy (HAART); unfortunately, these benefits have not extended equally to Blacks in the United States, possibly due to differences in patient comorbidities and demographics. These differences include rates of hepatitis B and C infection, substance use, and socioeconomic status. To investigate the impact of these factors, we compared hospital mortality and length of stay (LOS) between Blacks and Whites with HIV/AIDS while adjusting for differences in these key characteristics.

**Methods:**

The 1996–2006 National Hospital Discharge Surveys were used to identify HIV/AIDS patients admitted to US hospitals. Survey weights were incorporated to provide national estimates. Patients < 18 years of age, those who left against medical advice, those with an unknown discharge disposition and those with a LOS < 1 day were excluded. Patients were stratified into subgroups by race (Black or White). Two multivariable logistic regression models were constructed with race as the independent variable and outcomes (mortality and LOS > 10 days) as the dependent variables. Factors that were significantly different between Blacks and Whites at baseline via bivariable statistical tests were included as covariates.

**Results:**

In the general US population, there are approximately 5 times fewer Blacks than Whites. In the present study, 1.5 million HIV/AIDS hospital discharges were identified and Blacks were 6 times more likely to be hospitalized than Whites. Notably, Blacks had higher rates of substance use (30% vs. 24%; *P *< 0.001), opportunistic infections (27% vs. 26%; *P *< 0.001) and cocaine use (13% vs. 5%; *P *< 0.001). Conversely, fewer Blacks were co-infected with hepatitis C virus (8% vs. 12%; *P *< 0.001). Hepatitis B virus was relatively infrequent (3% for both groups). Crude mortality rates were similar for both cohorts (5%); however, a greater proportion of Blacks had a LOS > 10 days (21% vs. 19%; *P *< 0.001). Black race, in the presence of comorbidities, was correlated with a higher odds of LOS > 10 days (OR, 95% CI = 1.20 [1.10–1.30]), but was not significantly correlated with a higher odds of mortality (OR, 95% CI = 1.07 [0.93–1.25]).

**Conclusion:**

Black race is a predictor of LOS > 10 days, but not mortality, among HIV/AIDS patients admitted to US hospitals. It is possible that racial disparities in hospital outcomes may be closing with time.

## Background

Human Immunodeficiency Virus (HIV) has had a devastating impact on the United States, and the effects are far reaching. Each year in the United States, HIV infects over 56,000 people and claims upwards of 14,000 lives [1,2]. Despite these grim statistics, there has been considerable progress made in curbing the detrimental impact of the epidemic. The introduction of antiretroviral therapy in the late 1980s was the inaugural event in the therapeutic management of HIV patients, and led to lower rates of HIV-related morbidity and mortality [3]. Moreover, the advent of highly-active antiretroviral therapy (HAART) over a decade ago resulted in even greater reductions in HIV-related hospitalizations and mortality [4,5]. While HAART has decreased overall morbidity and mortality in the general HIV population, the benefits of these advances have not extended equally to the Black segment of the community [6-9].

Many Blacks may first seek care late in the course of their disease, after they have already become symptomatic with an opportunistic infection (OI) [10,11]. Because of this late diagnosis, they are at higher risk for an Acquired Immunodeficiency Syndrome (AIDS) diagnosis when they are found to be HIV positive, which may require immediate hospital admission [12]. Black patients may then experience longer hospitalizations and higher mortality rates than White patients [13-17]. These disparities in health outcomes between Black and White HIV patients may not be attributable to an imbalance in the receipt of HAART [18].

The presence of these comorbidities may worsen the overall health outcomes of those patients with advanced HIV/AIDS disease even when equivalent therapy is provided [18-22]. Substance use, hepatitis B virus (HBV), hepatitis C virus (HCV), and sexually transmitted infections (STIs) have all been linked to increased transmission of HIV, and can potentially complicate the course of the HIV disease [19,21,23-26]. These disparities between the Black HIV/AIDS population and the non-Black HIV/AIDS population are of critical importance; a deeper understanding of the impact of these disparities in Blacks is needed. To further this understanding, the present study evaluated health outcomes among HIV/AIDS patients in the 1996–2006 National Hospital Discharge Survey (NHDS). This study sought to: (1) compare health outcomes (mortality and length of hospital stay [LOS > 10 days]) between Black and White patients; (2) describe changes in health outcomes for Black and White patients over time in the HAART era; and, (3) quantify baseline rates of OIs, substance use, HBV, HCV, and STIs for Black and White patients.

## Methods

### Data source

Hospital discharge data were extracted from the 1996–2006 NHDS, a series of annual national surveys conducted by the National Center for Health Statistics (NCHS) of the Centers for Disease Control and Prevention (CDC) [27]. The NHDS has been conducted annually since 1965 by the NCHS. The NHDS are a series of voluntary nationwide sample surveys completed by short-stay hospitals in the United States. The NHDS collects information from non-federal hospitals (excluding military, Department of Veterans Affairs, and other federal facilities such as prison hospitals) in the United States. The US Bureau of the Census is the agent for data collection for these surveys. The hospital staff in each facility are trained by Census personnel to complete the survey instruments. The units of analyses are national discharges rather than estimates for individual patients. Some patients may have multiple discharges from a particular hospital in a given year and the possibility exists that they may be sampled more than once, but due to the complexity of the survey sampling, multiple discharges for an individual patient in a given year are unlikely. National estimates of diagnoses and procedures are classified by International Classification of Diseases, Ninth Revision, Clinical Modification (ICD-9) codes. The overall error rate for records manually coded is estimated at one percent for hospital diagnoses, 0.7 percent for surgical and diagnostic procedures, and 0.2 percent for non-medical data entry [27]. Data from the NHDS surveys has been used to evaluate other areas of racial disparities and hospital outcomes [28-30].

### Study definitions

An HIV/AIDS-associated hospitalization was defined as a hospitalization with at least one of the following ICD-9 codes for discharge diagnosis: 042-044 (HIV disease), V08 (asymptomatic HIV infection), or 079.53 (HIV-2 illness). ICD-9 codes were also collected for diagnoses of HBV, HCV, substance use, sexually transmitted infections, and OIs (Table 1). OIs were identified using the conditions included in the 1993 AIDS surveillance case definition [31]. Opportunistic infections included: candidiasis, cocciodomycosis, coccidiosis (in place of isosporiasis), cryptosporidiosis, cryptococcosis, cytomegalovirus disease, histoplasmosis, *Mycobacterium Avium*, pneumocystosis, progressive multifocal leukoencephalopathy, *Salmonella*, *Toxoplasmosis gondii*, and tuberculosis [31,32]. Sexually transmitted infections included chlamydia, gential herpes, gonorrhea, herpes simplex virus, human papilloma virus, Lymphogranuloma venereum, pelvic inflammatory disease, syphilis, and trichomoniasis. All opportunistic infections were classified as either preventable with chemoprophylaxis (*Mycobacterium Avium*, pnuemocystosis and *Toxoplasmosis gondii*) or non-preventable with chemoprophylaxis [33]. For the purpose of data analysis, collapsed categories were created for comorbidities that were reported infrequently. An "other substance use" category was created for types of substance use with a prevalence of < 1% and "other STI" for STIs < 1%.

**Table 1:** Codes for Diagnoses from International Classification of Diseases, 9th Revision, Clinical Modification (ICD-9-CM)

ICD-9-CM Code	Diagnosis
042, 044, 043, 079.53, V08	Human Immunodeficiency Virus/Acquired Immune Deficiency Syndrome

**Opportunistic Infections**	

112	Candidiasis

114	Coccidioidomycosis

007.2	Coccidiosis (for Isosporiasis)

007.4	Cryptosporidiosis

117.5	Cryptococcosis

078.5	Cytomegalovirus

115	Histoplasmosis

031.0, 031.2	*Mycobacterium avium*

136.3	Pneumocystosis

046.3	Progressive multifocal leukoencephalopathy

003	*Salmonella*

130	*Toxoplasmosis gondii*

010-018	Tuberculosis

**Hepatitis**	

070.41, 070.44, 070.51, 070.54, 070.7	Hepatitis C Virus

070.3, 070.2	Hepatitis B Virus

**Sexually Transmitted Infections**	

099.0	Chancroid

099.5	Chlamydia

054	Genital Herpes

098.11, V02.7	Gonorrhea

078.1,079.4, V73.81	Human Papilloma Virus

099.1	Lymphogranuloma venereum

614.9	Pelvic inflammatory disease

091, 092, 093, 094, 095, 095.7, 095.8, 095.9, 096, 097, 097.1, 097.9	Syphilis

131	Trichomoniasis

**Types of Substance Use**	

303, 305.0	Alcohol use

304.3, 305.2	Cannabis use

304.2, 305.6	Cocaine use

304.0, 305.5	Opioid use

305.1	Tobacco use

304.1, 305.4	Sedative, hypnotic, or anxiolytic use

304.5, 305.3	Hallucinogen use

305.7	Amphetamine or related sympathomimetic abuse

305.8	Anti-depressant type abuse

305.9	Other, mixed, or unspecified drug abuse

304.4	Amphetamine and other psychostimulant dependence

304.6	Other specified drug dependence

304.8	Combinations of drug dependence excluding opioid type drug

304.9	Unspecified drug dependence

304.7	Combinations of opioid type drug with any other

The NHDS surveys provide information about primary forms of hospital visit payment with standardized options. For the purposes of data analysis, the different forms of insurance were defined as uninsured (no charge or self-pay), government (Medicare, Medicaid, or other government form of payment), private (HMO/PPO, Blue Cross/Blue Shield, or other private insurance), and other/unknown (workers compensation, other, or not stated).

### Study design

This was a retrospective analysis of HIV/AIDS hospitalizations in the United States from 1996–2006. The University of Texas at Austin Institutional Review Board determined this protocol to be exempt (#2009-03-0100). Data included patient demographics (age, race, ethnicity, gender, and marital status), year of discharge, insurance status, geographic location within the United States, hospital LOS, and discharge status. Patients who were < 18 years, those who left against medical advice, those with an unknown discharge disposition, and those with a LOS < 1 day were excluded from data analysis. Patient discharge weighting was incorporated into the dataset to provide national estimates across the United States.

Hospital mortality and LOS > 10 days were evaluated. Mortality was classified as an inpatient admission resulting in death. In order to incorporate survey weights, LOS was then dichotomized (mean LOS ≤ 10 or >10 days) for all hospital discharges. This cut-off was chosen on the basis of the LOS distribution which demonstrated a peak of approximately 10 days. Chi-square tests were used to compare categorical baseline variables (survey year, age, gender, insurance status, and comorbid conditions) between races. Two multivariable logistic regression models were used to assess the impact of race (independent variable) on hospital mortality and LOS >10 days (dependent variables). Known predictors of poor health outcomes among HIV patients were entered simultaneously as covariates into both models. These included gender, insurance status, AIDS-related infections, and injection drug use [9,34,35]. The NHDS does not contain an ICD-9 code for 'injection drug use.' The most detailed ICD-9 code for heroin use was included under the umbrella heading of opioid use (which in addition to heroin also includes meperidine, morphine, and other opiates). To avoid grouping all types of substance abuse together, 'cocaine use' was considered a proxy for injection drug use. Likewise, variables that were statistically and clinically significant in chi-square analyses were also entered into both multivariable analyses as covariates. These variables included race, survey year, age, gender, insurance status, geographic region, presence of OI, HBV, HCV, and cocaine use. All data were analyzed using JMP 7.0^® ^(SAS Corp, Cary, NC) and an alpha-level of 0.05 was used to determine statistical significance.

## Results

From 1996–2006, 1.5 million HIV/AIDS hospital discharges were identified. On average, 138,016 hospitalizations occurred each year. The mean patient age was 42 years and 67% of all patients were male. Blacks accounted for more hospitalizations than Whites (62% vs. 38%). A large proportion of patients had government insurance (66%) or private insurance (22%). Bivariable analyses (Table 2) revealed a greater proportion of Blacks were female (41% vs. 21%; *P *< 0.001). Chi-square analysis revealed a significant relationship between insurance status and race (*P *< 0.001); Blacks had a higher likelihood of having some form of government insurance (70% vs. 58%) and fewer Blacks than Whites had private insurance (18% vs. 31%).

**Table 2:** Selected Discharge Characteristics among Blacks vs. Whites

Characteristic	Total (N = 1,518,173)	Race		P-Value
		Blacks (N = 948,666)	Whites (N = 569,507)	
**Survey Year**				***< 0.001***
1996	171,253	95,546(10%)	75,707 (13%)	
1997	133,493	78,665 (8%)	54,838 (10%)	
1998	140,619	82,730 (9%)	57,889 (10%)	
1999	125,398	78,075 (8%)	47,323 (8%)	
2000	119,673	76,629 (8%)	43,044 (8%)	
2001	136,796	93,424 (10%)	43,372 (8%)	
2002	134,593	83,645 (9%)	50,948 (9%)	
2003	146,975	94,379 (10%)	52,596 (9%)	
2004	149,817	92,883 (10%)	56,934 (10%)	
2005	121,116	81,165 (9%)	39,951 (7%)	
2006	138,440	91,525 (9%)	46,915 (8%)	

**Age (years)**				***< 0.001***
18–34	360,605	224,644 (24%)	135,961 (24%)	
35–49	869,517	542,349 (57%)	327,168 (57%)	
50–64	258,741	164,579 (17%)	94,162 (17%)	
≥ 65	29,310	17,094 (2%)	12,216 (2%)	

**Gender**				***< 0.001***
Male	1,014,194	562,565 (59%)	451,629 (79%)	
Female	503,979	386,101 (41%)	117,878 (21%)	

**Insurance Status**				***< 0.001***
Uninsured	135,124	88,389 (9%)	46,735 (8%)	
Government	994,492	662,787 (70%)	331,705 (58%)	
Private	341,686	167,102 (18%)	175,584 (31%)	
Other insurance	46,871	30,388 (3%)	16,483 (3%)	

**Any Opportunistic Infection (OI)**	406,661	255,988 (27%)	150,673 (26%)	***< 0.001***

**Preventable OI**	167,623	101,043 (11%)	66,580 (12%)	***< 0.001***

**Non-Preventable OI**	301,316	196,106 (21%)	110,210 (19%)	***< 0.001***

**Hepatitis B**	47,847	28,051 (3%)	19,796 (3%)	***< 0.001***

**Hepatitis C**	147,261	80,295 (8%)	66,966(12%)	***< 0.001***

**Substance Use**	421,591	285,133 (30%)	136,458 (24%)	***< 0.001***

**STIs**	79,243	54,123 (6%)	25,120 (4%)	***< 0.001***

### Comorbidities between Black and White patients

Substance use and OIs were present in 28% and 27% of all discharges, respectively; whereas HCV, STIs, and HBV were reported in 10%, 5%, and 3%, respectively. Common forms of substance use included cocaine use (9%), alcohol use (9%), opioid use (9%), and tobacco use (6%). Herpes simplex was not commonly reported (3%).

Results from bivariable analysis of characteristic differences between Blacks and Whites are reported in Table 2. A large proportion of both Black and White patients carried diagnoses for OIs (27% vs. 26%; *P *< 0.001). A significantly greater proportion of Blacks reported substance use (30% vs. 24%; *P *< 0.001). Conversely, fewer patients in the Black cohort were co-infected with HCV based on ICD-9 codes (8% vs. 12%;*P *< 0.001). The remaining comorbidities were relatively infrequent among Blacks and Whites. Further analysis revealed significant differences for comorbid subtypes between the two cohorts. Blacks, when compared to Whites, had a higher prevalence of non-preventable OI (21% vs. 19%; *P *< 0.001), cocaine use (13% vs. 5%; *P *< 0.001), opioid use (9% vs. 6%; *P *< 0.001), and alcohol use (10% vs. 8%; *P *< 0.001).

### Hospital mortality between Black and White patients

Overall, crude mortality rates were similar between Black and White cohorts 5% vs. 5% (OR 95% CI = 0.97 [0.96–0.99]). Simple logistic regression analysis revealed the following variables (*P *< 0.001) to be associated with mortality: race, survey year, age, gender, insurance status, presence of an OI, HBV, HCV, STIs and cocaine use. All clinically relevant variables from the regression analysis were subsequently entered in the multivariable model as covariates. Results from the multivariable analyses are presented in Tables 3 and 4. When significant predictors for mortality were entered into the model, Black race was not a significant independent predictor of mortality (OR, 95% CI = 1.07 [0.93–1.25], Table 3).

**Table 3:** Mortality Predictors from Multivariable Logistic Regression Analysis

Characteristic	OR (95% CI)	L-R Chi-Square	P-Value
**Survey Year**		68.2	***< 0.001***
1996	1.00 (reference)		
1997	1.02 (0.63–1.58)		
1998	1.25 (0.80–1.89)		
1999	1.05 (0.65–1.65)		
2000	0.74 (0.43–1.21)		
2001	1.19 (0.76–1.83)		
2002	0.98 (0.61–1.54)		
2003	1.94 (1.29–2.85)		
2004	0.59 (0.35–0.96)		
2005	0.37 (0.19–0.66)		
2006	0.64 (0.38–1.05)		

**Race**		0.9	0.3
White	1.00 (reference)		
Black	1.07 (0.93–1.25)		

**Age (years)**		12.8	***0.005***
18–34	1.00 (reference)		
35–49	0.66 (0.51–0.86)		
50–64	0.77 (0.55–1.08)		
≥ 65	3.06 (1.65–5.37)		

**Gender**		2.7	0.9
Female	1.00 (reference)		
Male	1.14 (0.98–1.34)		

**Insurance Status**		3.8	0.4
Uninsured	1.00 (reference)		
Government	0.81 (0.63–1.07)		
Private	0.76 (0.55–1.04)		
Other insurance	1.57 (0.85–2.74)		

Presence of Comorbidities*			

Preventable OI	2.39 (2.00–2.83)	86.9	***< 0.001***

Non-preventable OI	1.16 (0.98–1.37)	3.1	0.08

HBV	0.86 (0.54–1.31)	0.5	0.5

HCV	0.85 (0.64–1.11)	1.3	0.2

Cocaine use	0.46 (0.32–0.80)	25.2	***< 0.001***

*Reference group is the absence of a comorbidity where OR = 1.00

**Table 4:** Predictors for LOS > 10 days from Multivariable Logistic Regression Analysis

Characteristic	OR (95% CI)	L-R Chi-Square	P-Value
**Survey Year**		104.4	***< 0.001***
1996	1.00 (reference)		
1997	1.78 (1.41–2.25)		
1998	1.47 (1.17–1.86)		
1999	0.62 (0.47–0.81)		
2000	0.94 (0.72–1.22)		
2001	0.91 (0.71–1.17)		
2002	0.91 (0.71–1.17)		
2003	1.07 (0.85–1.36)		
2004	0.74 (0.58–0.95)		
2005	0.65 (0.49–0.86)		
2006	0.72 (0.56–0.93)		

**Race**		18.4	***< 0.001***
White	1.00 (reference)		
Black	1.20 (1.10–1.30)		

**Age**		26.4	***< 0.001***
18–34	1.00 (reference)		
35–49	0.98 (0.83–1.17)		
50–64	1.34 (1.09–1.64)		
≥ 65	1.10 (0.70–1.67)		

**Gender**		0.3	0.6
Female	1.00 (reference)		
Male	1.02 (0.94–1.11)		

**Insurance Status**		8.4	***0.04***
Uninsured	1.00 (reference)		
Government	1.22 (1.04–1.43)		
Private	0.96 (0.80–1.16)		
Other insurance	0.89 (0.62–1.27)		

Presence of Comorbidities*			

Preventable OI	1.97 (1.77–2.20)	142.9	***< 0.001***

Non-preventable OI	2.03 (1.86–2.22)	238.0	***< 0.001***

HBV	1.05 (0.84–1.31)	0.2	0.7

HCV	1.04 (0.90–1.19)	0.3	0.6

Cocaine use	0.98 (0.93–1.12)	0.1	0.8

*Reference group is the absence of a comorbidity where OR = 1.00

### Hospital LOS between Black and White patients

The proportion of patients with LOS > 10 days was higher for Blacks than Whites (21% vs. 19%; OR, 95% CI = 1.17 [1.16–1.18]). Simple logistic regression analysis revealed the following variables (*P *< 0.001) to be associated with LOS > 10 days: race, survey year, age, gender, insurance status, presence of OI, HBV, HCV, and cocaine use. All variables from the regression analysis were subsequently entered in the multivariable model as covariates. Multivariable analysis identified Black race as an independent predictor for LOS > 10 days (OR, 95% CI = 1.20 [1.10–1.30], Table 4).

### Annual hospital mortality and LOS trends between Black and White patients

**Figure 1:**
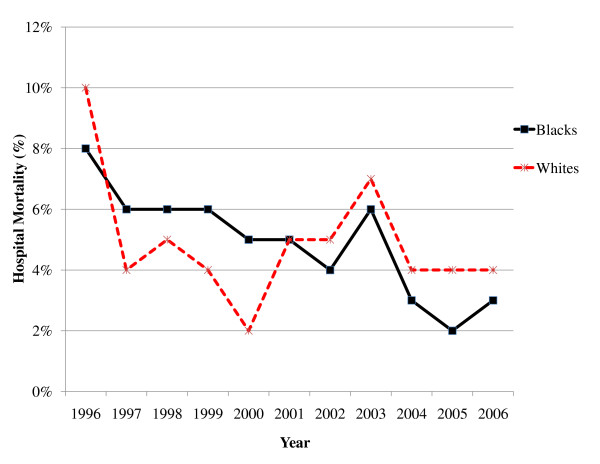
**Crude hospital mortality for Black and White HIV/AIDS patients from 1996–2006**.

**Figure 2:**
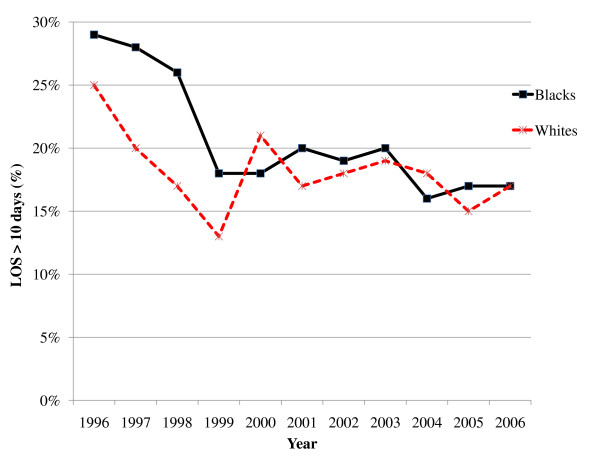
**Crude LOS > 10 days for Black and White HIV/AIDS patients from 1996–2006**.

**Figure 3:**
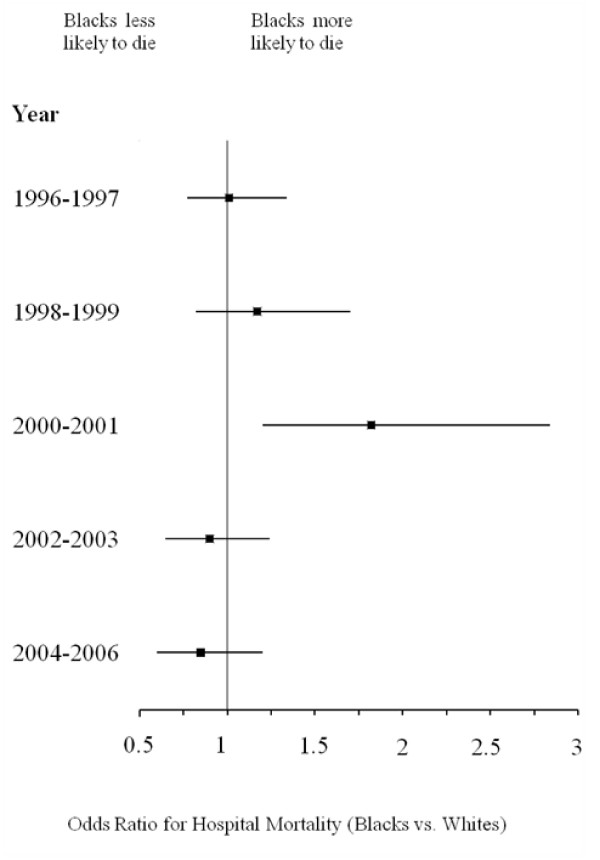
Adjusted hospital mortality for Black vs. White HIV/AIDS patients from 1996–2006 (Odds Ratio, 95% CI)*. *Odds ratios were calculated from the multivariable logistic regression model.

**Figure 4:**
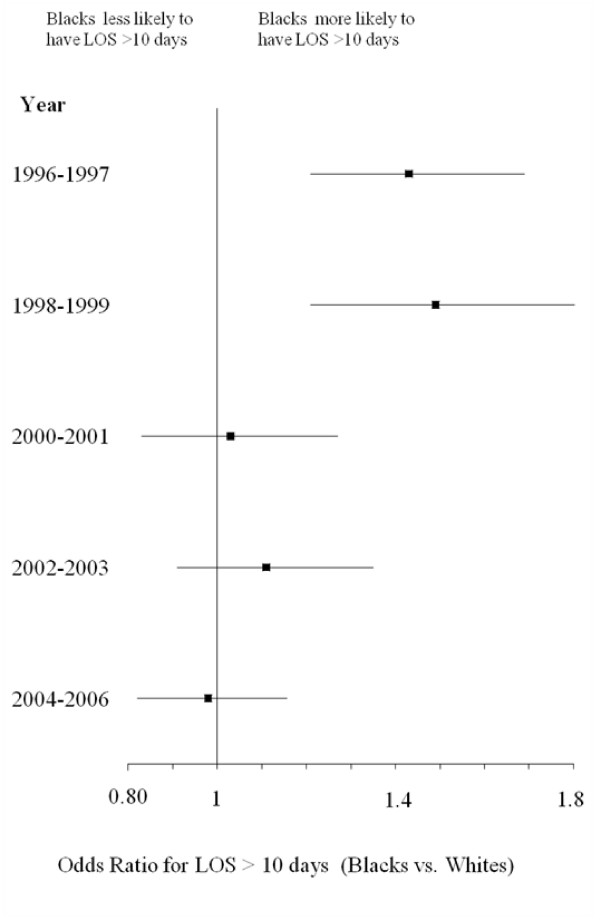
Adjusted LOS > 10 days for Black vs. White HIV/AIDS patients from 1996–2006 (Odds Ratio, 95% CI)*. *Odds ratios were calculated from the multivariable logistic regression model.

Figure 1 depicts trends in crude hospital mortality for Blacks and Whites from 1996–2006. Although both cohorts exhibited decreases in mortality, the decline for Blacks initially lagged behind that of Whites until 2001, when rates for mortality appeared to have converged between the two races. Crude rates for LOS > 10 days were greater for Blacks at the beginning of the study period, but the rates for the cohorts appeared to converge with time (Figure 2). Figures 3 and 4 reflect the adjusted hospital mortality and LOS trends over the last decade. Similar to the crude rates, the adjusted rates demonstrate the likelihood of hospital mortality and the likelihood for a longer LOS for Blacks were initially higher than that of Whites in 1996, but the odds for both outcomes appeared to decrease with time.

## Discussion

This study compared health outcomes for Black and White HIV/AIDS patients across the United States in the HAART era. Despite the many successes that have resulted from combating the disease over the past decade, published data suggest that Blacks continue to experience a disproportionate burden of HIV infection. Not only are Blacks overrepresented in the total number of people living with the disease, they continue to experience the highest rates of new infections and AIDS-related deaths [36]. In the general US population, there are approximately 5 times fewer Blacks than Whites. Of the 1.5 million HIV/AIDS hospital discharges identified in this study, Blacks were 6 times more likely to be hospitalized than Whites, a disparity that has been identified in previous studies conducted after the initiation of HAART [16,17,37]. Although the crude mortality rates were similar for Blacks and Whites in the present study, Black race, in the presence of comorbidities, substance use, and socioeconomic status, was associated with longer LOS, but not in-hospital mortality. Furthermore, both mortality and LOS seem to have converged with time. Based on the multivariable regression models, it is possible that other demographic factors including comorbidities and insurance status may be more predictive of outcome than simply race.

Although HAART is clearly an effective treatment for HIV, the clinical benefits of therapy may not extend equally to patients who are diagnosed with advanced stages of infections [4,5]. Thus, the high proportion of patients with OIs was concerning for both study cohorts. Nonetheless, Black patients demonstrated slightly higher rates of OIs compared to Whites. This study confirmed that the presence of an OI was predictive of higher mortality as well as extended hospitals stays in multivariable regression models. As previously mentioned, Blacks have been reported to seek care later in the course of infection after they are symptomatic from their illness [13-15]. The increased rate of mortality among Blacks has been well documented [9,16,23,38]. One such study by Jain and colleagues reported poorer outcomes among patients with OIs, specifically highlighting the elevated risk for Black patients [34]. The Jain study differs from the present study in that the investigators were unable to evaluate comorbidities, and various forms of substance use.

Baseline rates of substance use were higher among Blacks than Whites. The rate of cocaine use for Blacks was almost three times that of Whites; interestingly, regression analysis revealed cocaine use was 'protective' for mortality, perhaps because cocaine use was also associated with younger age. Nevertheless, studies suggest illicit drug use may have a negative impact on the treatment and course of HIV/AIDS infection [39,40]. Concomitant use of illicit substances may also interfere with a patients' ability to adhere to antiretroviral regimens, thereby placing them at risk for poorer health outcomes [23,39,40]. Cocaine has been demonstrated *in vitro *and in experimental animal models to enhance viral replication and interfere with the body's ability to defend itself naturally against infection. This may hasten disease progression and the development of AIDS-defining conditions, even among patients who are adherent to antiretroviral therapy [41-43]. However, definitive data directly implicating illicit drug use as a causative factor in increased HIV susceptibility or disease progression remain elusive [41]. Of the various types of substance use in this study, cocaine use was the most likely form of injection drug use, which has also been implicated in the transmission of not only HIV, but also HBV and HCV, particularly among minority populations [23-25].

Beyond the HIV/AIDS population, other racial disparities exist for Blacks; they tend to have higher rates of HBV and HCV compared to Whites [24,25,44]. Furthermore, response to therapy for HCV is markedly diminished for Blacks [45]. Interestingly, in the present study the rates of HBV coinfection were similar among both study cohorts and HCV coinfection rates were lower for Blacks than for Whites. Although hepatitis coinfection is associated with increased mortality, the true rates of HIV/HCV coinfection and its implications for health outcomes among Black populations remain ill-defined [26,46].

Likewise, the national impact of race disparities among individuals with concomitant STIs is not well-characterized [47]. The increased risk for contracting HIV with simultaneous STIs (both ulcerative and non ulcerative) has been well established [47-50]. HIV, coupled with infections such as syphilis, may negatively impact the immune status of the individual and may lead to higher rates of treatment failure [19,51,52]. Furthermore, genital herpes, gonorrhea, chlamydia, syphilis, bacterial vaginosis and chancroid, are reportedly higher in minority populations [47]. In the present study, reported rates of STIs were relatively low in both groups of hospitalized patients. Complications from STIs including severe herpes simplex virus, syphilis with neurologic involvement, and acute pelvic inflammatory may require aggressive treatment necessitating hospitalization, whereas most other STIs can be managed effectively in the ambulatory setting [53]. Therefore, STIs were not included in regression analyses. Definitive conclusions regarding STIs could not be made from this cohort of hospitalized patients. Nevertheless, a higher percentage of Blacks carried an STI ICD-9 diagnosis.

A surprising, but fortunate finding from this study is the possibility that racial disparities in hospital outcomes may be closing with time. This possible reduction in racial disparities in outcome has been demonstrated elsewhere [8,37,54,55]. Similar to the Giordano and Hlaing studies, this study was not able to incorporate antiretroviral use in the regression analysis [37,55]. Antiretrovirals, if prescribed properly, may reduce differences in outcomes between Blacks and Whites [8,54]. Yet, in stark contrast, Jain and colleagues concluded that the disparity for higher mortality for Black HIV patients has actually *widened *in the HAART era [34]. According to their results, the elevated risk of a poor outcome was also attributable to the presence of an opportunistic infection, a finding that is consistent with the present study. Another study by Levine and colleagues also substantiates these findings. They identified that higher rates of mortality existed among Black males both before and after the introduction of HAART [9]. The investigators lacked data for comorbid conditions and also did not evaluate hospitalized patients. The current study evaluated all-cause hospital mortality, whereas the two studies that concluded Blacks were at increased risk for mortality used surveillance data and a combination of inpatient and outpatient data [56,57]. The present study had the most patient records and the longest duration of any of the studies identified in the literature review.

This study shares the limitations of all retrospective cohort studies. In addition, the analysis was limited by the lack of available clinical variables, including medication information, time of HIV diagnosis, and objective clinical markers of disease progression. Therefore, we could not assess the relationships between quality of care indicators and patient outcomes. Additionally, management of acute HBV, HCV, and STIs may not require hospitalization. These data are also limited by proxies for racism and discrimination that are known predictors of racially-biased outcomes. These variables are important when describing disparities and could have at least partly explained the racial variations and/or non-variations found in this study. The data source lacks information regarding patients of Hispanic descent; therefore, we were unable to draw conclusions about health disparities for this minority group that is also facing a disproportionate burden of HIV [36]. Another limitation is that patients who were admitted for less than one day, or who left the hospital against medical advice were excluded from the analysis. It is possible these patients ultimately suffered suboptimal health outcomes (early mortality). The survey scope encompassed only non-federal hospitals, so the results may not be applicable to HIV/AIDS patients admitted to other US hospitals, such as the Veterans Healthcare Administration. Finally, several other complex socioeconomic issues that may affect health outcomes were not evaluated in this study.

## Conclusion

This study characterized the rates of comorbid conditions, hospital mortality and LOS, and evaluated trends in health outcomes among Black and White HIV/AIDS patients over a decade. Some disparities may still persist despite the availability of effective interventions to reduce morbidity and mortality. This national assessment of hospital discharges demonstrates that Black race may be predictive of longer LOS, but not mortality. In addition, disparities in health outcomes may be decreasing with time. Further research efforts are warranted to recognize and clarify the complexities facing the Black HIV/AIDS population in order to reduce health disparities.

## Abbreviations

HAART: highly active antiretroviral therapy; HIV: Human Immunodeficiency Virus; AIDS: Acquired Immunodeficiency Syndrome; LOS: length of hospital stay; CI: confidence interval; OR: odds ratio; L-R likelihood ratio; OI: opportunistic infection; STI: sexually transmitted infection.

## Competing interests

The authors declare that they have no competing interests.

## Authors' contributions

CUO had full access to the data in the study and assisted with the study design, study concepts, data analysis and interpretation, and drafting of the manuscript. CRF directed and supervised all aspects of the study from its conception to the finalization of the manuscript. JMH assisted in drafting the manuscript. JS assisted in the statistical analysis and interpretation of data, as well as critical revision of the manuscript for important intellectual content. All authors assisted with critical revision of the manuscript for important intellectual content, and have approved the final manuscript.

## Pre-publication history

The pre-publication history for this paper can be accessed here:

http://www.biomedcentral.com/1471-2334/9/127/prepub
